# The Impact of E-Learning on Adherence to Guidelines for Acute Gastroenteritis: A Single-Arm Intervention Study

**DOI:** 10.1371/journal.pone.0132213

**Published:** 2015-07-06

**Authors:** Emanuele Nicastro, Andrea Lo Vecchio, Ilaria Liguoro, Anna Chmielewska, Caroline De Bruyn, Jernej Dolinsek, Elena Doroshina, Smaragdi Fessatou, Tudor Lucian Pop, Christine Prell, Merit Monique Tabbers, Marta Tavares, Pinar Urenden-Elicin, Dario Bruzzese, Irina Zakharova, Bhupinder Sandhu, Alfredo Guarino

**Affiliations:** 1 Department of Translational Medical Science, Sector of Pediatrics. University Federico II, Naples, Italy; 2 Paediatric Hepatology Gastroenterology and Transplantation Unit, Hospital Papa Giovanni XXIII, Bergamo, Italy; 3 Department of Pediatrics, The Medical University of Warsaw, Warsaw, Poland; 4 Universitair Ziekenhuis Brussel Kinderen, Vrije Universiteit Brussel, Brussels, Belgium; 5 University Medical Centre, Maribor, Slovenia; 6 Russian Medical Postgraduate Academy, Moscow, Russian Federation; 7 Third Department of Pediatrics of University of Athens, Greece; 8 2nd Pediatric Clinic, Iuliu Haţieganu, University of Medicine and Pharmacy, Cluj-Napoca, Romania; 9 Dr. von Hauner Children’s Hospital, University of Munich Medical Center, Munich, Germany; 10 Emma's Children's Hospital Academic Medical Centre, Amsterdam, Netherlands; 11 Department of Pediatrics, Hospital Sao Joao, Porto, Portugal; 12 Department of Pediatrics, Marmara University, Istanbul, Turkey; 13 Department of Public Health, University of Naples “Federico II,” Italy; 14 Department of Paediatric Gastroenterology, Bristol Royal Hospital for Children, Bristol, United Kingdom; Emory University School of Medicine, UNITED STATES

## Abstract

**Objective:**

E-learning is a candidate tool for clinical practice guidelines (CPG) implementation due to its versatility, universal access and low costs. We aimed to assess the impact of a five-module e-learning course about CPG for acute gastroenteritis (AGE) on physicians’ knowledge and clinical practice.

**Study design:**

This work was conceived as a pre/post single-arm intervention study. Physicians from 11 European countries registered for the online course. Personal data, pre- and post-course questionnaires and clinical data about 3 to 5 children with AGE managed by each physician before and after the course were collected. Primary outcome measures included the proportion of participants fully adherent to CPG and number of patients managed with full adherence.

**Results:**

Among the 149 physicians who signed up for the e-learning course, 59 took the course and reported on their case management of 519 children <5 years of age who were referred to their practice because of AGE (281 and 264 children seen before and after the course, respectively). The course improved knowledge scores (pre-course 8.6 ± 2.7 versus post-course 12.8 ± 2.1, P < 0.001), average adherence (from 87.0 ± 7.7% to 90.6 ± 7.1%, P = 0.001) and the number of patients managed in full adherence with the guidelines (from 33.6 ± 31.7% to 43.9 ± 36.1%, P = 0.037).

**Conclusions:**

E-learning is effective in increasing knowledge and improving clinical practice in paediatric AGE and is an effective tool for implementing clinical practice guidelines.

## Introduction

Clinical practice guidelines (CPG) are systematically developed statements that assist healthcare practitioners in making decisions about appropriate care for specific diseases based on evidence [1,2]. The overall quality of CPG is increasing, and their development, quality control, and evaluation protocols are well established [3,4]. A major limitation of guidelines is the challenge of implementation [4]. Implementing recommendations in clinical practice is a complex and largely uncharted process [5]. Traditional implementation methods such as conferences, web sites and didactic lectures may fail because they are time-consuming and expensive, especially for the practitioner [6,7]. In addition, there is little evidence of the impact that traditional implementation methods have on clinical practice, because outcome measures are usually knowledge based rather than reflective of actual changes in clinical behaviours [8].

Acute gastroenteritis (AGE) is a major problem in paediatrics. CPG for AGE are available, but poorly applied, and many children receive unnecessary interventions [9,10]. This leads to an excess of referrals, hospitalizations, and drug prescriptions, which ultimately results in higher costs and minimal patient benefit [11]. According to a recently published study, more than 50% of children presenting with AGE were admitted without meeting the guideline criteria for hospitalization [12]. Low adherence to recommendations for AGE treatment has been reported in both developed and developing countries [13,14]. Compliance with recommendations may improve children’s clinical outcomes and reduce complications and costs [8]. Given low guideline compliance, combined with a high prevalence and burden of illness, AGE is an ideal candidate for CPG implementation through e-learning. Recently, the Federation of the Societies of Paediatric Gastroenterology Hepatology and Nutrition (FISPGHAN) identified e-learning programs as an educational priority that should be exploited to decrease AGE-related morbidity and mortality worldwide [15].

E-learning has been proposed as a tool for continuing education in medical science, and results have been promising [16,17]. However, the potential use of technology in medical education and the transfer of knowledge to practice has not been fully exploited [18]. We assessed the impact of an e-learning course on the management of AGE in European children based on the CPG jointly produced by the European Society for Paediatric Gastroenterology, Hepatology and Nutrition (ESPGHAN) and the European Society for Paediatric Infectious Diseases (ESPID), followed as standard of care by all the participating countries [19]. Both the knowledge and clinical practice of European paediatricians and general practitioners were evaluated.

## Methods

### Study design

This study was a pre/post single-arm intervention study. The experimental phase, including the baseline record and the post-data collection, was carried out from May 20th to September 30th, 2013. However, the entire project, including the e-learning course design and production, and the dataset analysis, required about 14 months (September 2012 to November 2013).

### Participants

A total of 415 physicians from 11 European countries were invited to participate in the study. A tutor for each country was identified from among the members of the Scientific Committee of the Tutorial European Electronic Network on Acute Gastroenteritis (TEEN-AGE) project, in order to assist physicians in the recruitment process and resolve technical problems technical problems related to the United European Gastroenterology (UEG) website. Each tutor was asked to identify at least 25 physicians from his/her country to be invited to participate. To obtain a randomly enrolled sample from each country and to minimize selection bias, participating physicians were identified through regional and national databases or through national scientific societies.

No specific inclusion criteria were applied beyond the comprehension of English and the ability to use a computer.

### E-learning course design and production

The e-learning course included five learning modules addressing the five key areas of AGE management based on ESPGHAN/ESPID guidelines: 1) Introduction and definitions, 2) Clinical assessment and management, 3) Oral rehydration and active treatment, 4) Other treatments, and 5) Treatment of inpatients. All of the learning materials (video, slides, evaluation questionnaires, figures, web references, checklists) were reviewed and approved by the Scientific Committee of the TEEN-AGE initiative for content and format. The course is freely available, in English, and only requires the physician to register online (https://www.ueg.eu/education/courses/online-courses/acute-gastroenteritis/) [20].

### Intervention

After registering on the UEG website, each participant received a personal code to access the section containing the e-learning course and the patient data portal.

All participating physicians were asked to provide their personal profile information (age, country, languages spoken, previous experience with e-learning) and other information about their practice (specialty, years of activity, inpatient/outpatient work setting). They completed a baseline and post-course questionnaire measuring their knowledge of AGE and its treatment, which included questions from a large pool of calibrated items that had been previously assessed for reliability and content validity.

At baseline, each physician also reported on his/her case management of 3 to 5 consecutive patients <5 years of age and referred for AGE, defined as a decrease in the consistency of stools (loose or liquid) and/or an increase in the frequency of evacuations (>3 in 24 hours) with or without fever or vomiting^19^. Each learner recorded clinical case information at the end of the child’s visit or, for inpatients, at the time of discharge. These cases were entered in an anonymous electronic Case Report Form (CRF). The CRF included 5 domains: child and family data, clinical features, home management, reasons for admission, and hospital management. Additionally, any underlying chronic conditions and/or concomitant acute illnesses were recorded in the CRF to aid in the interpretation of outcomes according to case-specific risk factors.

After completing the baseline phase, physicians had one month to view the five learning modules. Subsequently, they were asked to load information on another 3 to 5 consecutive cases of AGE using the same CRF. The post-course test of knowledge was the final measure.

### Definition of inappropriate interventions

Inappropriate interventions in the management of AGE were identified by comparing the reported medical interventions, including prescriptions and procedures, with the CPG recommendations in each of the following domains: evaluation of the main signs/symptoms, concordance between the objective assessment of dehydration and the physician’s estimate, nutritional interventions, requests for blood tests, rehydration route, prescription of microbiological investigations, and prescription of probiotics, antiemetics, antibiotics, and other anti-diarrheal drugs (S1 Table). The same methodology for the assessment of the appropriateness of medical interventions has been used in a previous publication^12^.

Inappropriate interventions were divided into major and minor violations. A major violation was defined as a) a medical intervention inconsistent with CPG recommendations that might negatively affect the course of the disease and/or might be associated with unnecessary costs (such as the use of antiemetics specifically contraindicated or not considered as appropriate treatment according to the guidelines—e.g., Metoclopramide and Domperidone), or b) any violation to “high grade” recommendations in the guidelines (CPG recommendations supported by level I or II evidence according to the Muir-Gray score). A minor violation was defined as a) an intervention that did not substantially change the outcome but was generally considered inappropriate (such as the prescription of probiotics for which the current available evidence of efficacy is not conclusive–e.g., weakly recommended probiotics), or b) any violation to “low grade” recommendations in the referral guidelines (level III, IV, and V evidence according to Muir-Gray).

To produce a quantitative estimate of adherence to the AGE CPG in this study, any major violation reduced the overall adherence by 10% and any minor violation by 5%; the final percentage score was calculated by summing the results reported for each domain, with a maximum of 100%. Scores >90% were considered full adherence.

Primary outcomes of the e-learning intervention were the proportion of participants whose medical interventions were fully adherent to guidelines and the scores on the knowledge questionnaires (number of correct answers out of a total of 15 questions). The amount of time taken to complete the knowledge test was also recorded in the e-learning platform as indirect proof of improved knowledge.

### Ethical considerations

The study was approved by the Education Committee of ESPGHAN and conducted with the technical partnership of the UEG as part of the TEEN-AGE initiative. All participants signed written informed consent forms.

### Statistical analysis

Statistical analyses were performed in the statistical computing environment R (version 3.0.1; R Foundation for Statistical Computing, Vienna, Austria). Data for continuous variables are expressed as means ± SD. Data for categorical variables are presented as frequencies and percentages. Pre- and post-course differences in the theoretical knowledge of CPG recommendations and average adherence scores were evaluated using the Wilcoxon signed-rank test for paired samples.

To examine the impact of physician- and patient-related factors on adherence, a two-level random intercept multilevel logistic regression analysis (MLRA) was used to account for the clustering of AGE cases among physicians. MLRA was conducted separately for the pre-education patient group (PreEG) and post-education patient group (PostEG) data to investigate whether factors associated with non-adherence to CPG prior to the e-learning course were consistent with inappropriate interventions after the course. Adjusted odds ratios and corresponding 95% confidence intervals were obtained using the MLR method.

All tests were two-tailed, and p values <0.05 were considered significant.

## Results

**Fig 1:**
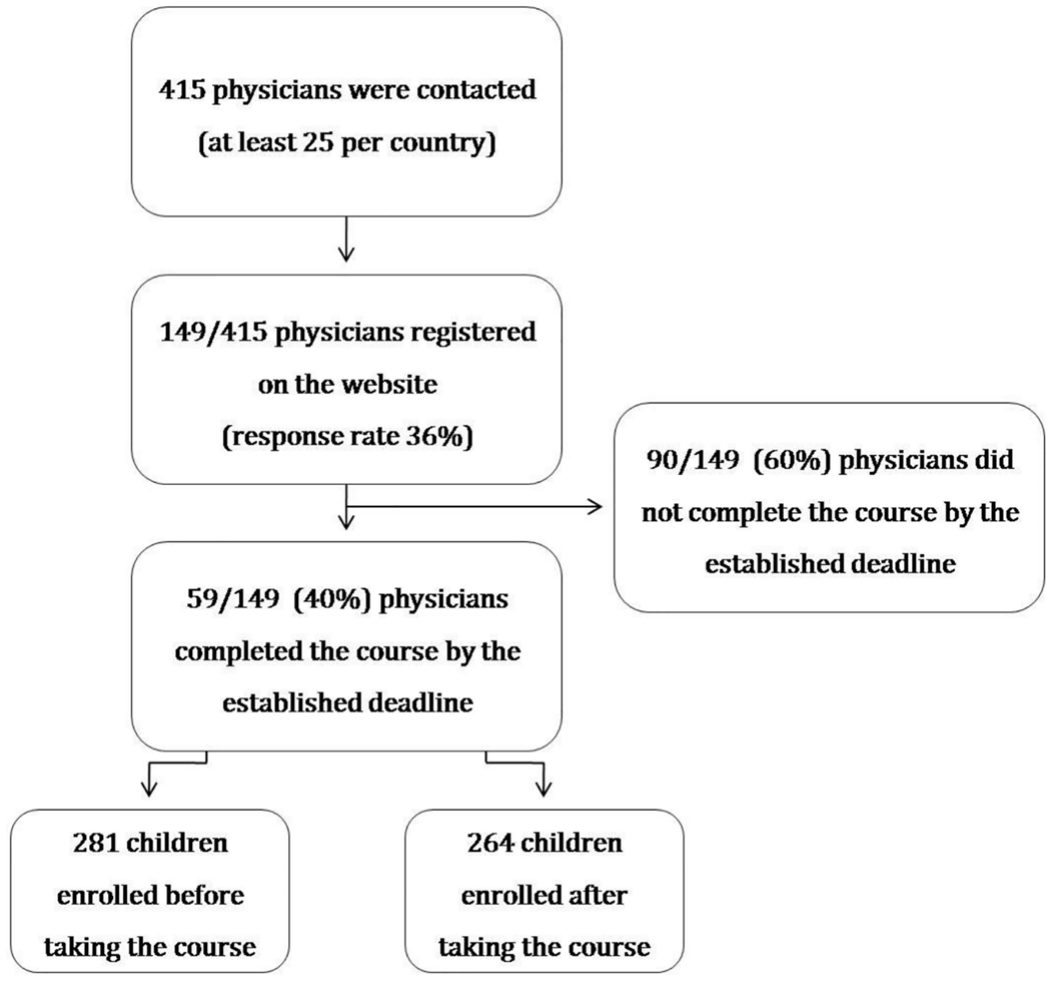
Study design: flowchart of the participants.

Out of the 415 practitioners contacted, a total of 149 physicians registered for the e-learning course (response rate 36%). Fifty-nine (40%) of physicians (45 females, median age 40 years, range 26–59) who registered for the course completed it before the deadline (Fig 1); their baseline characteristics are listed in Table 1. Participants were from Slovenia (12); Greece (11); the Netherlands (9); Portugal, Romania, and Russia–Moscow Region (5 each); Turkey and Italy (3 each); and Poland, Belgium, and Germany (2 each). Fifty-six out of 59 (95%) were pediatricians, while 3/59 (5%) enrolled physicians declared to be general practitioners who worked in pediatric setting. No differences in age, gender, years of practice, setting of practice, previous experience with e-learning, or previous knowledge of CPG were observed between the physicians who completed the course and those who did not.

**Table 1:** Baseline characteristics of the enrolled physicians.

Characteristic	
Mean age in years ± SD	39.5 ± 7.57
Number of Male/Female (%)	14/45 (24)
Less than 10 years of practice: n/N (%)	27/59 (46)
At least 10 years of practice: n/N (%)	32/59 (54)
Inpatient setting: n/N (%)	39/59 (66)
Outpatient setting: n/N (%)	20/59 (34)
Previous experience with e-learning: n/N (%)	32/59 (54)
Previous awareness of ESPGHAN guidelines: n/N (%)	34/59 (57)

The data of 545 children with AGE (249 females; median age 21 months, range 1–60) were registered by the participants, 281 before (PreEG, 51%) and 264 after taking the course (PostEG, 49%). Three hundred and forty-eight patients (64%) were managed in a hospital setting, and 197 (36%) were treated in an outpatient setting. A total of 25 out of 545 children (5%) presented with severe dehydration according to physician estimates. Specific clinical characteristics of the children reported on in the PreEG and PostEG assessments are shown in Table 2.

**Table 2:** Characteristics of the children with AGE registered as clinical cases.

	PreEG	PostEG	
	n = 281	n = 264	P
	n/N (%)	n/N (%)	
M/F	152/129	144/120	1
Mean age ± SD (months)	23.04 ± 15.46	23.38 ± 16.24	0.98
Weight-for-age (mean ± SD)	0.03 ± 0.9	0.029 ± 0.092	1
Inpatients	184/281 (65)	164/264 (62)	0.423
Outpatients	97/281 (35)	100/264 (38)	0.423
Chronic underlying disease	20/281 (7)	25/264 (9)	0.352
Concomitant acute illness	35/281 (12)	32/264 (12)	0.054
ORS at home	128/281 (46)	148/264 (56)	0.0164
Children with severe dehydration	13/281 (5)	12/264(5)	0.2860

Pre-EG = pre-education group; PostEG = post-education group; ORS = oral rehydration solution.

**Fig 2:**
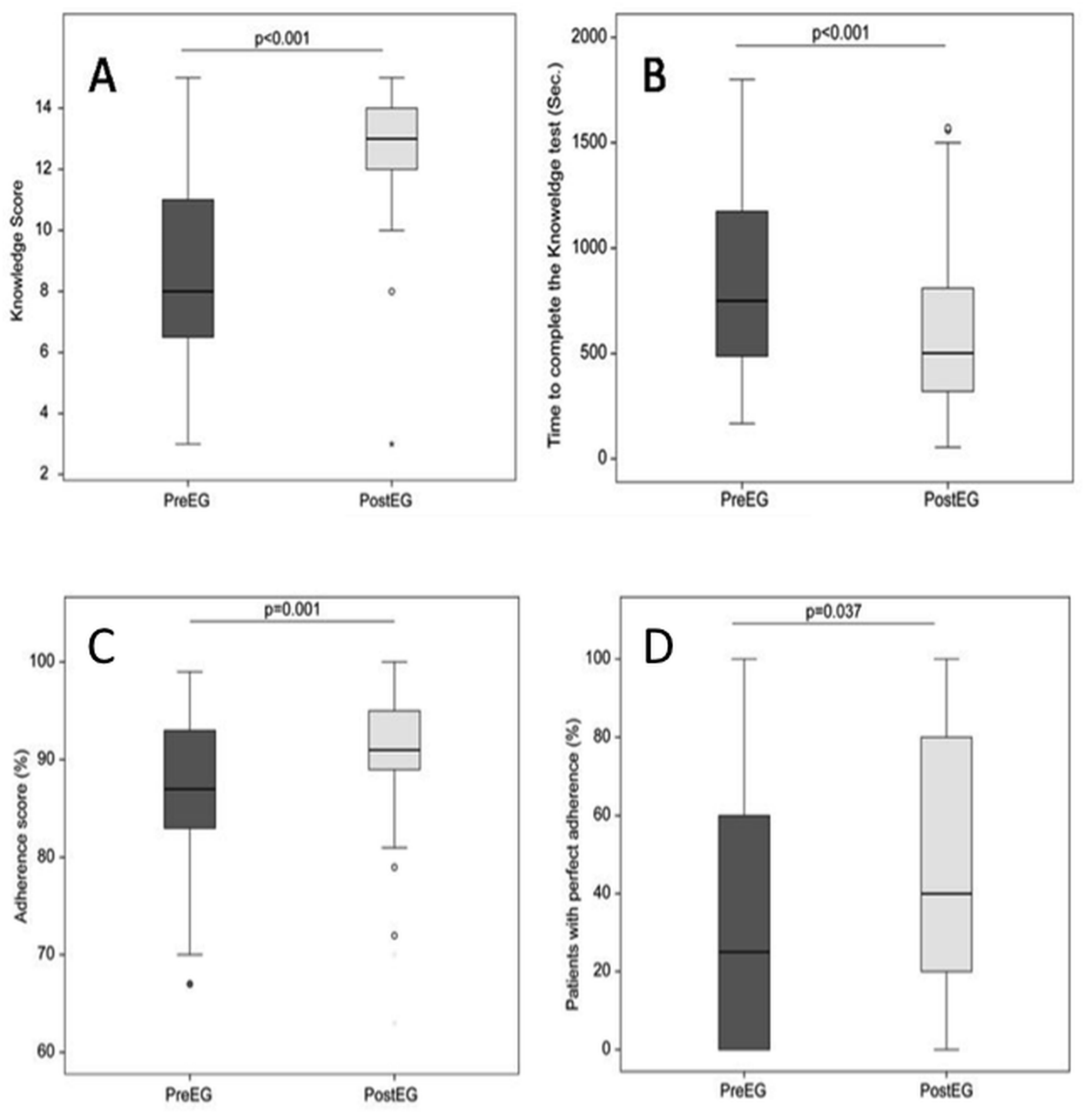
Impact of e-learning on knowledge and clinical practice about the management of acute gastroenteritis in children before (Pre) and after the e-learning intervention (Post). A) learners’ scores, and B) time to complete the 15-question evaluation tests (as recorded by the e-learning platform), C) average adherence percentage score in 545 children <5 years managed before (Pre) and after (Post) the e-learning corse, D) proportion of patients managed in full adherence with the guidelines.

Knowledge about the CPG on AGE treatment increased after the e-learning course, based on the scores on the 15-question (1 point per question) knowledge test before (8.6 ± 2.7 points) and the course (12.8 ± 2.1 points, P <0.001). The response time also decreased after the course (878 ± 503 versus 579 ± 379 seconds, P <0.001) for the 59 physicians who completed the study (Fig 2).

**Fig 3:**
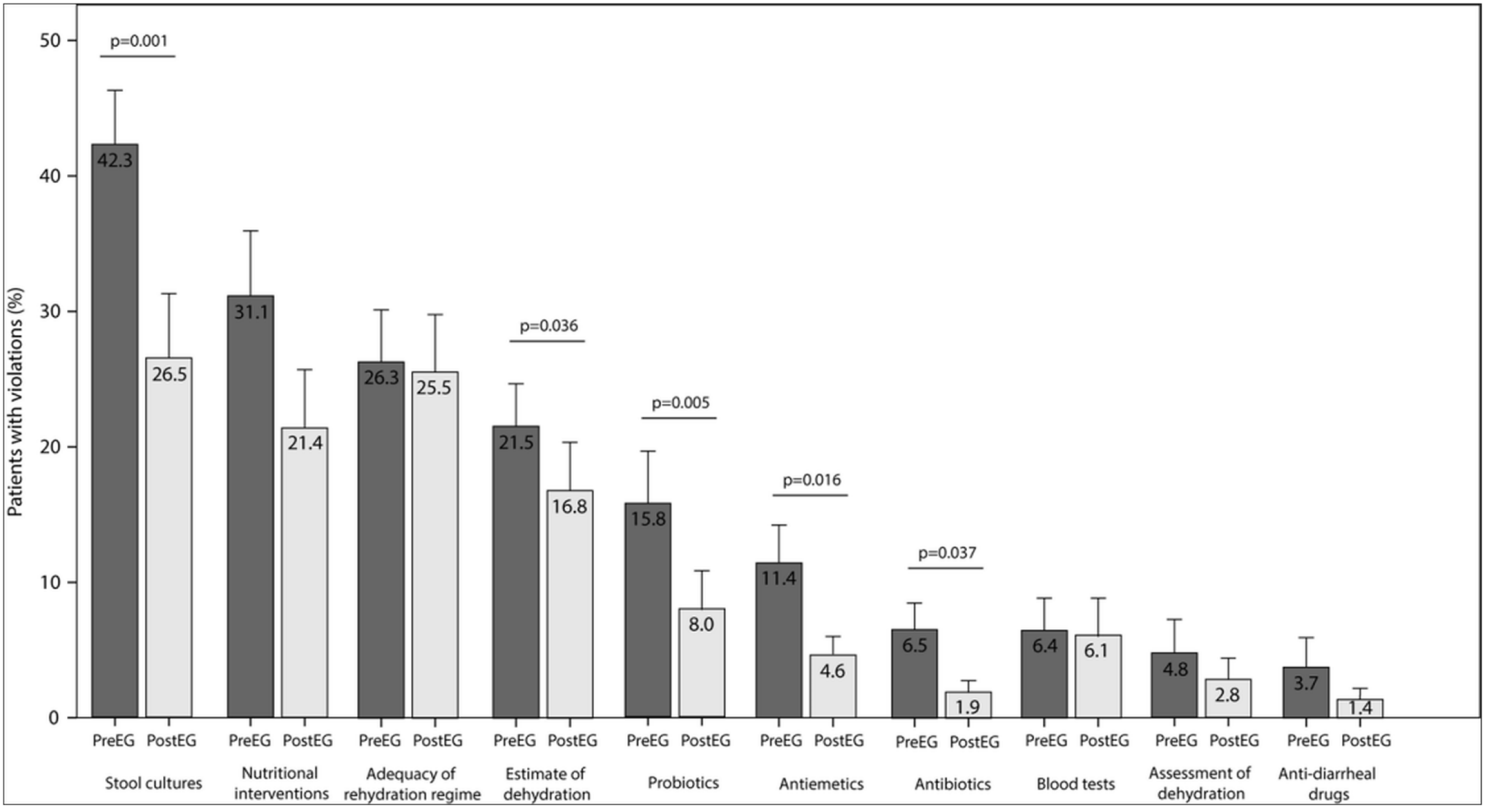
Changes in inappropriate interventions for acute gastroenteritis in children <5 years managed before (Pre) and after (Post) e-learning.

The proportion of patients managed in full adherence with the guidelines (i.e., no inappropriate interventions or only one minor violation) increased from 33.6 ± 31.7% to 43.9 ± 36.1% (P = 0.037). Similarly, the average adherence score increased from 87.0 ± 7.7% to 90.6 ± 7.1% (P = 0.001) (Fig 2). The proportion of patients who received inappropriate interventions in each domain was calculated. The most common violations to the CPG were orders for stool cultures in the absence of appropriate indications. Unnecessary dietary changes and inconsistent estimates of dehydration compared to objective parameters were also frequently observed. As shown in Fig 3, the e-learning course reduced inappropriate interventions in all of these domains. We also observed a non-significant trend toward a reduction in inappropriate nutritional interventions (P = 0.055). In all, 22% of patients in the PreEG were inappropriately admitted to the hospital, compared to 15% in the PostEG (P = 0.200) patients. The proportion of hospitalized children with moderate or severe dehydration (indicated by >5% weight gain at discharge) was similar in both groups (25% in the PreEG and 26.5% in the PostEG; P = 0.841).

We assessed the link between specific physician- and patient-related factors and discrepancies with the guideline recommendations. We also investigated whether these factors were still associated with inappropriate interventions after the course. No differences in age, gender, years of practice, setting of practice (outpatient vs inpatient), previous experience with e-learning were found between physicians who were adherent to CPG and those who weren’t (S2 Table). These findings are similar before and after the e-learning course. Before the course, physicians who were unaware of the guidelines were less likely to adhere to the CPG (OR = 0.29; 95% CI [0.10 to 0.86]; P = 0.026), while no difference was found after (OR = 1.92; 95% CI [0.58 to 6.37]; P = 0.289). In terms of clinical characteristics, children in the PreEG with bloody diarrhoea (OR = 5.75 95% CI [1.39 to 23.89]; P = 0.016) or abdominal pain (OR = 1.88; 95% CI [1.1 to 3.24]; P = 0.02) were more likely to receive inappropriate interventions at baseline (OR = 1.9; 95% CI [0.46 to 7.84], P = 0.37), but this increased risk disappeared after the course (OR = 0.61; 95% CI [0.251 to 0.49], P = 0.279). In contrast, frequent vomiting episodes (>5/day) remained associated with inappropriate management before (OR = 4.07; 95% CI [1.39 to 11.89]; P = 0.01) and after the course (OR = 5.22; 95% CI [1.64 to 16.69]; P = 0.005). Patients in the PreEG with chronic diseases were more likely to have any inappropriate interventions (OR = 0.24; 95% CI [0.07 to 0.86]; P = 0.028) than those in the PostEG.

## Discussion

The e-learning course examined in this study increased physicians’ theoretical knowledge about the appropriate management of AGE. Translated into clinical practice, better knowledge should reduce the time to effective intervention, which provides a benefit in the physician’s daily practice. The overall adherence to CPG improved, as illustrated by a statistically significant increase in mean adherence score and consistent reduction in several inappropriate management interventions.

E-learning is an useful strategy to improve medical practice due to its universal availability, asynchronous accessibility, interactivity, integration of implementation tools (including checklists and web references.), and low cost for the learner [21,22]. However, evidence of its efficacy in improving clinical practice is lacking, since it has been mostly applied in undergraduate medical education setting [23–27]. Many studies have used surrogate outcomes to predict changes in practice, such as drug and prescription test [16], simulations of resuscitation procedures [17], and structured clinical examination tests [28]. Several e-learning programs were developed recently in Europe, with variable results [29–32]. In the UK, the online interactive Diabetes Needs Assessment Tool (DNAT) has been found to produce significant improvements in knowledge and changes in clinical practice [25]. The Interactive Spaced Education, an online-asynchronous method for e-learning, has been shown to improve adherence to CPG for prostate specific antigen screening and to improve the clinical care and outcomes in patients with hypertension [33–34]. This confirms that online learning for doctors can be effective, at least for some specific content.

Although e-learning applications appear to be a “smart tool” for medical education, the acceptance of this approach is still poor, as suggested by the dropout rate in this study. It should be noted that the limited time allotted to complete the course and the baseline case registration requirement were the major causes of dropout, as reported by the participants who evaluated the e-learning course at the end of the modules, but these limitations were related to the structure of the study. However in the first six months after publication on the website, over 500 practitioners all around Europe took the e-learning course without taking part to the “experimental phase”, indicating that most physicians are interested in educational initiatives but the inclusion as active part of a trial may potentially limit their commitment. On the other hand, the role of the tutor was a positive determinant of course completion, as full assistance was guaranteed for every kind of technical and support need. Tutors also sent weekly reminders to those who had not completed the course.

We evaluated the determinants of the specific discrepancies in the application of CPG through a logistic regression model and examined the role of both physician and patient factors. The e-learning course closed the gap between physicians who had previous knowledge of CPG and those who did not. The presence of abdominal pain and bloody diarrhoea were major determinants of non-adherence before the e-learning intervention, but this changed after the course, indicating that the intervention reduced mismanagement triggered by clinically alarming signs.

We also investigated the types of inappropriate interventions that were used and measured the effectiveness of medical practice in specific areas. Notably, a decrease in violations was observed after the e-learning course in all domains. E-learning education was highly effective in reducing inappropriate requests for microbiological investigations. The course also reduced dietary changes, improved the estimate of the degree of dehydration, decreased the use of disapproved probiotics, and lowered the inappropriate use of antiemetics and antibiotics. A major exception to this success was that the presence of frequent vomiting in children with AGE continued to be associated with unnecessary interventions after the course. Antiemetics were not recommended in the 2008 AGE guidelines, but are still a matter of debate. Certain guidelines suggest a possible positive role of antiemetics, including ondansetron, in paediatric emergency departments to reduce the number of hospital admissions [35–38]. Other guidelines, including ESPGHAN/ESPID, are more conservative, based on an FDA warning about the potentially severe side effects of ondansetron [39].

The assessment of dehydration in children with AGE is the key step for diagnosis and treatment, and drives medical behaviours. According to guidelines the best estimate of AGE severity is the degree of dehydration [19]. The concordance between physicians’ estimate of dehydration grade and the objective assessment was fair, meaning that several physicians overestimated the grade of dehydration. Such a discrepancy in estimates of dehydration could have impacted adherence scores thus affecting the interpretation of the effect of the e-learning course. A similar incongruity between guidelines and practice was evident in a previous Canadian study that reported a moderate-to-severe dehydration in only 2% of children with gastroenteritis although 30% of them received intravenous rehydration [40].

This study has some limitations. Firstly, the participating physicians were from various different countries, thus results may have been affected by country-related factors. However, we did not focus on specific country-related differences either because the samples of each country were too small to be compared, or because the etiology, clinical features and target population, as well as the relative management of AGE may be considered similar all over Europe. Also, with regard to timing, AGE has changes in seasonal incidence and the study fell in a period of low incidence [41]. Therefore, the CPG adherence may be altered by the relative frequency with which physicians had diagnosed AGE. This choice was due to the deadlines imposed by the educational grant. Moreover, since physicians reported on their own practice, a recall bias is a potential risk; although all participants were asked to record the data at the end of the visit or discharge, the time between the patient visit and data entry was not registered in the system. In addition, the awareness of taking part in an intervention study on the management of AGE may have affected the physicians’ behaviours, independently of their knowledge improvement, resulting in a potential Hawthorne-like effect and in a desirability bias. Furthermore, there were no controls in place to prevent physicians from selecting cases which they knew were managed in accordance with guidelines. Consecutively selected charts helped mitigate this potential bias, but considering the short duration of the post intervention phase and the limited number of patients per physician, it is conceivable that physicians could have picked all of the patients they know they managed well shortly after completing the course. However, any quality improvement intervention is aimed at improving clinical practice starting from robust evidence of efficacy. The e-learning course was based on the guidelines published by the ESPGHAN/ESPID in 2008 and actively distributed and presented in many different scientific and social settings. In most of cases this kind of intervention do not include a control group, even if the presence of controls may be helpful in demonstrating a “potential improvement” in knowledge and practice in those physicians not exposed to this educational instrument. Relevant changes in medical practice in accordance to recommendations available since about 5 years seemed unlikely and, in addition, physician enrolled in the study worked as “self-control”. Self-report mechanism has also some merits to be acknowledged, such as efficient resource utilization and the educational value of auditing one's own practice, which is pervasive in quality improvement and implementation science. It is also well known that practically all pre-post study improves knowledge in short term, and it is important to do a follow up after a reasonable time, to see the retention of knowledge.

However, this is the first study to directly assess the impact of e-learning education on paediatric practice; the results clearly demonstrate that this tool is effective not only in improving knowledge about CPG, but also in increasing the concordance between recommended and actual practice.

As in other areas of information technology and web-based education, large scale and targeted information is needed to attract the interest of the desired audience. These successful results have led ESPGHAN, in close collaboration with UEG, to implement its e-learning program for use in education and training in Europe; the organization is also establishing a dedicated editorial project to shape specific competencies in delivering widespread, high quality education in paediatric gastroenterology, hepatology, and nutrition.

## Supporting Information

S1 Table Major and minor violations to guidelines recommendations. (DOCX)

S2 Table MLRA model for the estimate of the risk of non-perfect adherence. (DOCX)
