# Supplementary material for: The Impact of E-Learning on Adherence to Guidelines for Acute Gastroenteritis: A Single-Arm Intervention Study
Source: PLoS One. 2015 Jul 6;10(7):e0132213. doi: 10.1371/journal.pone.0132213 (PMC4493016; doi:10.1371/journal.pone.0132213)
Supplement: S1 Table — (DOCX) [file pone.0132213.s001.docx]

**S1 Table. Major and minor violations to guidelines recommendations.**

| **Guidelines domain** | **Violations to guidelines recommendations** | |
| --- | --- | --- |
|  | **Major** | **Minor** |
| **Clinical evaluation** | | |
| **Assessment of the degree of dehydration** | Physician did not report any of the signs/symptoms of dehydration: capillary refill time, skin turgor, thirst, respiratory pattern, urinary output, child’s general appearance | Physician reported less than 50% of reliable signs/symptoms of dehydration  (capillary refill should be reported) |
| **Physician’s estimate of dehydration** | / | Physician’s estimate of dehydration at the child’s admission did not reflect the grade of dehydration based on more reliable signs/symptoms |
| **Diagnostic work up** | | |
| **Blood tests** | Did not require electrolyte evaluation in children undergoing blood tests | Prescription of blood tests other than electrolytes, CBC, and CRP in otherwise healthy children |
| **Stool culture** | / | Stool culture in otherwise healthy children without risk factors or overt bloody diarrhoea |
| **Treatment** | | |
| **Rehydration regime** | Exclusive oral rehydration in hospitalised children | / |
| **Nutritional interventions** | - Withdrawal > 6 hours - Cessation of breast feeding - Lactose-free formula - Cow‘s milk based protein free formula - Sports drinks | Any elimination diet (e.g., the BRAT diet) |
| **Anti-emetics** | Antiemetic drugs specifically contraindicated or not considered as appropriate treatment according to the guidelines  (e.g., Metoclopramide and Domperidone) | Antiemetic drugs prescribed in the absence of intractable or persistent vomiting or when oral rehydration failure was not reported |
| **Probiotics** | Probiotics prescribed but strain or product not reported | Any probiotic strains different from those recommended by the guidelines  (*LGG* and *S. boulardii*)  Probiotics for which evidence of efficacy is not conclusive |
| **Anti-diarrheal drugs** | All drugs other than those considered as appropriate treatment according to the guidelines (Smectite, Zinc, Racecadotril/Acetorphan) | / |
| **Antibiotics** | Any antibiotic prescribed without a positive culture or a declared risk factor  Treatment of non-typhi Salmonella in healthy children | Treatment of Campylobacter > 48-72 hours after the onset of disease |
