# Supplementary material for: The Impact of E-Learning on Adherence to Guidelines for Acute Gastroenteritis: A Single-Arm Intervention Study
Source: PLoS One. 2015 Jul 6;10(7):e0132213. doi: 10.1371/journal.pone.0132213 (PMC4493016; doi:10.1371/journal.pone.0132213)
Supplement: S2 Table — (DOCX) [file pone.0132213.s002.docx]

**S2 Table.** **MLRA model for the estimate of the risk of non-perfect adherence**

|  | **PreEG** |  | **PostEG** |  |
| --- | --- | --- | --- | --- |
|  | **Odds Ratio [95% CI]** | **P** | **Odds Ratio [95% CI]** | **P** |
| ***Physician characteristics*** | | | | |
| Gender (F vs M) | 0.77 [0.23 to 2.59] | 0.674 | 0.6 [0.15 to 2.41] | 0.472 |
| Age (Years) | 1.04 [0.95 to 1.15] | 0.404 | 1.02 [0.9 to 1.14] | 0.776 |
| Previous experience with e-L | 1.26 [0.44 to 3.62] | 0.67 | 1.67 [0.5 to 5.62] | 0.406 |
| Years of activity (≥10 vs <10) | 0.62 [0.16 to 2.33] | 0.477 | 0.21 [0.04 to 1.12] | 0.068 |
| Specialty (Paediatrician vs GP) | 1.01 [0.17 to 6.05] | 0.992 | 1.73 [0.19 to 16.23] | 0.63 |
| Setting (Outpatient vs Inpatient) | 0.56 [0.18 to 1.81] | 0.335 | 0.31 [0.08 to 1.18] | 0.086 |
| Previous knowledge of CPG | **0.29 [0.1** to **0.86]** | **0.026** | 1.92 [0.58 to 6.37] | 0.289 |
| ***Patients characteristics*** | | | | |
| Gender (F vs M) | 1.1 [0.58 to 2.11] | 0.765 | 0.88 [0.43 to 1.8] | 0.728 |
| Age (Months) | 1 [0.97 to 1.02] | 0.844 | 1.01 [0.98 to 1.03] | 0.66 |
| Chronic disease (yes vs no) | **0.24 [0.07** to **0.86]** | **0.028** | 0.92 [0.22 to 3.82] | 0.911 |
| Concomitant acute illness (yes vs no) | 1.2 [0.43 to 3.35] | 0.725 | 1.55 [0.48 to 5.05] | 0.465 |
| Episodes of vomiting |  |  |  |  |
| 3 to 5 vs. <3 | 1.2 [0.52 to 2.74] | 0.668 | 0.44 [0.17 to 1.14] | 0.092 |
| >5 vs. <3 | **4.07 [1.39** to **11.89]** | **0.01** | **5.22 [1.64** to **16.69]** | **0.005** |
| Abdominal pain (yes vs no) | **1.88 [1.1** to **3.24]** | **0.022** | 0.61 [0.25 to 1.49] | 0.279 |
| Diuresis (decreased vs normal) | 0.83 [0.37 to 1.86] | 0.651 | 0.68 [0.27 to 1.69] | 0.403 |
| Duration of symptoms (days) | 1.13 [0.92 to 1.37] | 0.24 | 1.14 [0.91 to 1.43] | 0.248 |
| Stool output |  |  |  |  |
| 3 to 5 vs. <3 | 0.96 [0.37 to 2.54] | 0.942 | 2 [0.71 to 5.66] | 0.191 |
| >5 vs. <3 | 1.36 [0.5 to 3.75] | 0.548 | 0.79 [0.25 to 2.48] | 0.688 |
| Bloody diarrhoea (yes vs no) | **5.75 [1.39** to **23.89]** | **0.016** | 1.9 [0.46 to 7.84] | 0.377 |

Pre-EG = pre-educational group; PostEG = post-educational group; GP = general practitioner.
